# Communication and Support‐Focused Experiences and Needs of Young Adult Couples Coping With Cancer: A Qualitative Study

**DOI:** 10.1002/pon.70248

**Published:** 2025-07-30

**Authors:** Katie Darabos, Sharon L. Manne, Katie A. Devine, Sean McHugh, Shannon Desbiens

**Affiliations:** ^1^ Rutgers School of Public Health Piscataway New Jersey USA; ^2^ Rutgers Cancer Institute New Brunswick New Jersey USA

**Keywords:** cancer, communication, oncology, qualitative interview, social support, young adult couples

## Abstract

**Background:**

Cancer among young adult (YA) couples is a profoundly distressing experience, extracting a significant toll on YA couples' relationships (e.g., with partners, family, friends). It is well recognized that effective communication within social relationships is critical for fostering well‐being among couples coping with cancer. Despite this, limited research exists focused on communication and support needs among YA couples.

**Aims:**

Through qualitative semi‐structured interviews, we explored the unique needs and experiences of YA couples with a focus on communication and support within their relationship and with their social support networks.

**Methods:**

Fifteen semi‐structured interviews were conducted with YA couples. YA survivors were eligible if they were diagnosed with cancer as a young adult (aged 18–39) within the past 5 years. Relevant themes in literature guided the development and use of semi‐structured interview guides.

**Results:**

Content‐structuring qualitative analysis was used to identify themes. Across 15 YA couples, four main themes emerged: dyadic communication, dyadic relationship changes, social support network challenges and opportunities, and resource needs.

**Conclusions:**

Results highlight YA couples' communication and support needs that are central, and external, to the couple. These findings elucidate how YA couples navigate the cancer experience together, highlighting experiences, needs, and concerns that are central to the developmentally off‐time nature of cancer during their relationship. Additionally, results have implications for intervention development focused on communication and support among this vulnerable and underrepresented YA couple population.

## 1 Background

Cancer is often considered a “we disease” impacting the patient, their partner, and their relationship [1]. Population estimates suggest that close to two‐thirds (62%) of the 80,000 young adults (YAs; aged 18–39) diagnosed with cancer each year in the United States identify as being in a committed, romantic relationship [2]. For young adult couples coping with cancer, managing the diagnosis and treatment of cancer in one partner is a profoundly stressful experience. Relative to older couples coping with cancer (aged ≥ 40), YA couples coping with cancer face unique challenges to timely accomplishment of normative life milestones (e.g., marriage, family planning, parenthood). Importantly, well‐documented disparities among YA survivors as compared with older survivors, particularly with regard to financial hardship, psychological distress, and long‐term recurrence and late effect risks can lead to persistent long‐term stress [3].

These stressors can extract a significant toll on YA couples' relationships. They experience poorer relationship satisfaction, elevated levels of psychological distress, and have an increased risk of divorce/separation as compared with older survivor couples [2, 4, 5, 6]. Even with high pre‐cancer relationship quality, the challenging treatment and post‐treatment landscape among YA couples may contribute to ongoing stress long after the cancer diagnosis [7]. As survivors of YA cancer often live decades beyond their diagnosis [8], it is crucial to understand how to promote well‐being in this vulnerable YA couple population.

For older adult couples, an extensive literature has documented couples' communication strategies that are associated with distress and relationship satisfaction [1, 9]. One key finding is that couples' ability to communicate constructively (self‐disclosure, perceived responsiveness to disclosure) is associated with better relationship satisfaction and psychological adjustment [9, 10, 11, 12]. Emerging evidence suggests that, compared to older adult couples, YA couples engage in fewer positive communication to manage complex stressors and are more likely to engage in negative communication (e.g., holding back, criticism) [4, 6]. These data indicate that challenges around communication and support may be unique to one's stage of life [6]. Thus, the existing adult cancer literature may not meet the needs of YA couples, who are often in the early years of their relationship and less experienced in navigating the challenges associated with cancer [13].

In addition to relying on one another, YA couples depend on a wide range of support from members of their social support networks (e.g., family, friends) to navigate challenges [14, 15]. Social connectedness (connections/relations with others) is central to providing a continuous foundation across the cancer continuum and serves to maintain a sense of normalcy among YA couples [16]. However, this may be challenging for YA couples who are often leaning on their family and friends as a unit for the first time, navigating family dynamics, and figuring out their joint needs and concerns. As YA couples are more likely to depend on their immediate family and friends for guidance and support [17, 18, 19], understanding how relationships function within a broader social network with its own unique stressors that impact that coupe relationship is critical [20].

Decades of research grounded in social support and stress buffering highlight the influential role of communication and support on a myriad of relational and health outcomes [21, 22, 23, 24, 25]. However, how YA couples communicate and support one another during the cancer experience is not well understood in the literature. Even less is known about YA couples interactions within their social networks. Existing research among YA couples is not dyadic. Rather, studies have separately focused on each partners' perspectives on the same types of experiences [16, 26, 27, 28, 29, 30]. In sum, there is a gap in knowledge on YA couples' joint experiences, needs, and concerns. Accordingly, the aim of the current study is to identify and describe themes of young adult couples' communication‐ and support‐focused experiences, needs, and concerns both within (dyadic) and outside (support network) their relationship.

## 2 Methods

The consolidated criteria for reporting qualitative research (COREQ) checklist was followed in reporting the methods and findings of this study [31].

### 2.1 Participants

Semi‐structured in‐depth qualitative dyadic interviews were conducted with YA couples (*N* = 15 couples). Eligible YAs and their partners were recruited over the phone from a large East Coast Cancer Center in the United States. YA survivors were identified via chart review and were eligible if they were currently between the ages of 18–39, diagnosed with cancer as a YA (between the ages of 18–39) within the past 5 years, and identified as being in a committed, romantic relationship for at least 1 year. To account for potential age differences in couples (e.g., YA survivor 38, YA partner 41), partners of YA survivors were eligible if they were currently between the ages of 18–45. However, all partners must have been between the ages of 18–39 at the time of the YA survivors' cancer diagnosis. YA couples were compensated with a $100 gift card for their participation. All interviews were conducted in English. All procedures were approved by the Institutional Review Board (Approval: Pro2023000562).

### 2.2 Semi‐Structured Interviews

Following completion of informed consent, semi‐structured interviews were conducted via videoconference in July‐November 2024. Interviews lasted approximately 60 min (*M* = 63.3, SD = 17.6, range = 30–98 min) and were video‐ and audio‐recorded. Interviews were conducted with both partners together because of the shared nature of the conversations that can often elicit deep discussion of experiences, needs, and concerns, especially for sensitive topics like fertility and mortality. Semi‐structured interviews were conducted by trained members of the research team (K.D., S.M., S.D.) using the same standardized, semi‐structured interview guide.

All interviews used a semi‐structured format following a pre‐specified interview guide designed to facilitate discussion and examination of young adult couple couples' communication‐ and support‐focused experiences, needs, and concerns both within and outside their relationship. The interview guide was informed through conceptual models, including dyadic coping [32] and the interpersonal process model of intimacy [33], through relevant themes identified in the dyadic and social support literature, and through input from authors [4, 5, 18, 34]. Specifically, we focused on shared concepts within these models and existing research such as disclosure, supportive and unsupportive reactions, and coping. To facilitate rapport and to get an introduction to the couples' cancer story, the qualitative guide opened with general relationship questions (e.g., how long have you been together, do you have any children) and an opportunity to share how the YA survivor was diagnosed. From there questions were broken into two main parts: (1) Dyadic Communication and Support, and (2) Social Support Network Communication and Support. Sample questions included: *Are there any cancer‐related topics that you (or your partner) found difficult to talk together about? Can you tell me about a time when your interactions or discussions with members of your* *support network felt different because of your (partners) cancer diagnosis*? (see Supplemental Material for the Interview Guide). Demographic data (e.g., age, race/ethnicity) were collected at the end of each qualitative interview. Clinical data (e.g., cancer type, treatment, time since diagnosis) were collected via the medical chart review.

### 2.3 Data Analysis

Semi‐structured interviews were transcribed verbatim and imported into ATLAS.ti. Transcripts of semi‐structured interviews were analyzed by trained members of the study team (K.D., S.M., S.D.) using the content‐structuring qualitative analysis method according to Kuckartz, which structures, analyzes, and interprets the data on the basis of deductively and inductively formed main and subcategories across several steps [35]. While approaches to coding are similar, in content‐structuring analysis frequency is a key factor for drawing meaningful conclusions as it reveals patterns and trends within the entire data set, whereas thematic analysis focuses on the underlying meaning or deeper significance of the data. First, members of the coding team engaged in an initial reading of the transcripts, marking specific segments of text based on common themes in the YA and couple support literature [18, 19] and through similarities across interviews. A preliminary list of codes, informed deductively from theoretical frameworks and previous research [4, 5, 18, 34], was agreed upon by the coding team. From this, thematic main categories were developed. Each transcript was then independently coded by the study team with these main categories. Afterwords subcategories were determined inductively. To ensure rigor of analysis, half of the transcripts were double coded independently by a trained member of the study team to ensure that there was no drift. The full study coding team met regularly. Discrepant codes were resolved through discussion. For this study, saturation was achieved when little additional information was gained from subsequent transcripts. We used thematic coverage of higher‐order groupings (identified themes) across the transcripts to determine saturation [36].

## 3 Results

### 3.1 Sample

A total of 15 couples participated. Demographic and medical characteristics (YA survivor) of the study sample are displayed in Table 1. On average, YA survivors were 34 years of age (SD = 3.4) and most identified as male (53%) and non‐Hispanic white (60%). YA survivors were on average 2 years from diagnosis (*M* = 23.4 months, SD = 14.6 months) with a range of cancer diagnoses represented (e.g., breast, lymphoma, testicular, thyroid). YA partners were, on average, 34 years of age (SD = 4.9) and most identified as female (53%) and non‐Hispanic white (80%). YA couples reported being together an average of 11 years (SD = 5.5 years). Nine (60%) couples were married, with length of marriage on average 6 years (SD = 3.1 years). One couple identified as the same sex. All couples reported being together during cancer diagnosis and treatment.

**TABLE 1:** Participant demographics and medical characteristics (*N* = 15 dyads).

Variable	YA survivor	YA partner
	*N* (%) or *M* (SD)	
Age (years)	33.9 (3.4)	34.4 (4.9)
Gender		
Male	8 (53.0%)	7 (47.0%)
Female	7 (47.0%)	8 (53.0%)
Hispanic		
Race		
Non‐hispanic white	9 (60%)	12 (80%)
Non‐hispanic black or African American	2 (13%)	2 (13%)
Hispanic	2 (13%)	1 (7%)
Other	2 (13%)	0 (0%)
Cancer diagnosis, *N* (%)		
Breast or gynecological	3 (20.0%)	—
Leukemia/Lymphoma	3 (20.0%)	—
Testicular	3 (20.0%)	—
Other^a^	6 (40.0%)	—
Cancer stage		
Early stage (stage I‐III)	10 (66.7%)	—
Metastatic (stave IV)	3 (20%)	—
Unstaged	2 (13.3%)	—
Treatment received^b^		
Surgery	11 (73.3%)	—
Chemotherapy	8 (53.3%)	—
Radiation	2 (13.3%)	—
Treatment status		
On active treatment	6 (40%)	—
Not on active treatment	9 (60%)	—
Time since diagnosis (months)	23.4 months (14.5)	

	Relationship characteristics	
Relationship length (years)		
Couples that reported being married (*n* = 9)	6.1 (3.1)	
Couples that reported not being married (*n* = 6)	7.4 (4.2)	
Number of children		
0 (or not mentioned)	9 (60.0%)	
1 or 2	4 (27.0%)	
3 or more	2 (13.0%)	

^a^ Other cancer diagnoses: small intestine, sarcoma, skin, thyroid, nasopharynx.

^b^ Survivors could have received more than one treatment.

### 3.2 Overview of Qualitative Findings

Four themes emerged from the analysis: (1) Dyadic Communication, (2) Dyadic Relationship Changes, (3) Social Support Network Challenges and Opportunities, and (4) Resource Needs. Main themes, corresponding subthemes, and illustrative quotes are shown in Table 2. For the purposes of verbatim quotes, we have provided the age and sex of the YA survivor or YA partner and how long they have been in a relationship or married at the time of the interview (< 5 years, > 5 years). With 15 qualitative interviews among YA couples, full thematic coverage was achieved.

**TABLE 2:** Illustrative quotes by theme and endorsement (%) by subtheme.

Theme 1: Dyadic Communication
Positive communication (*n* = 14 couples; 93%)
“I would say that our communication got better over time. But it wasn't the best that it could be at the beginning, but who knows how to deal with something like that until you're going through it, you know? That's something that we had to learn to deal with and work through. At the beginning, it was like a bomb that went off in this relationship, where we had to figure out how to talk to each other about this and what we can talk to each other about, because certain things bothered him, and certain things bothered me and it was like a—I wouldn't say like a clash, but we had to figure out how our two styles of communicating about cancer would work for us.” (37‐year‐old male survivor; married < 5 years)
Negative communication (*n* = 10 couples; 67% of couples)
“There's plenty of fights and stress and yelling and hanging up fueled by not feeling good, fueled by the stress, me being stressed about, you know, am I gonna die, fueled in a negative head space, fueled in [partners'] stress balancing normal life while having to take on the weight of my diagnosis.” (24‐year‐old male survivor; in a relationship for < 5 years)
Difficult topics for conversation (*n* = 9 couples; 60% of couples)
Fertility (*n* = 6 couples; 40% of couples)
“At the time we were going through everything with the cancer, we were not intending on having kids. And then in the couple of years after that, we have had kind of a change and have been going down that path of trying. So at the time, it didn't really matter to me, but now it is kind of like, oh, I probably should've known more or heard more about—my doctors say, oh, this won't impact your fertility. And it may just have been me not digging because kids weren't on the table at that time. But, I think that kind of thing in some hindsight now is kind of, that would be good to know of, how does this affect me, and are there lasting effects or not, especially now as we go through infertility. But I think that's a topic that I think is probably, especially in young adults, an important one.” (36‐year‐old male partner; married > 5 years)
Mortality (*n* = 5 couples; 33% of couples)
“There are morbid things that I fear, like fearing death and going into hospice and that, like the painful part of cancer. I don't feel necessarily comfortable talking about that with [partner] because it's such a heavy topic and it tends to bring us down, so like I reserve that for my therapist, but we communicate about everything else cancer related, symptom management, treatments, what we think is the best solution, how we can proceed like through the rest of our lives.” (24‐year‐old male survivor; in a relationship for < 5 years)
Financial (*n* = 3 couples; 20% of couples)
“I just wanted to make sure that if I was to pass, that I would leave my husband with some money and leave him with a business that was thriving in some type of aspect, so that he would be financially good and that he could, in turn, take care of my mother and my family. So that was just a rough process for me.” (37‐year‐old male survivor; married < 5 years)

**Theme 2: Dyadic relationship changes**
Shift in pre‐cancer responsibilities (more than half of couples)
*Survivor*: “At the end of the first cycle, our washer decided to—our pump—it's downstairs. Our sump pump exploded and flooded our laundry room. So I'm there, like, lightheaded trying to drink some gatorade, but bending over to try to sop up water in our laundry room because—and then we had a guy come over to help look at all the stuff, and I'm literally just laying on the couch, like, hey, like, I'm just a waste of life over here.” *Partner*: “But that's—I could see where it got to the point that his body was taxed, and he couldn't do stuff anymore … so I was like, okay, well, I guess I'll be taking on everything. And I understood that, but it worked out.” (35 year old male survivor, 37 year old female partner; married > 5 years).
Strengthening of the relationship (more than half of couples)
“I would say we were incredibly strong before this. I would say we're equally, if not way more, stronger after this. I think we've navigated—we're navigating it very well. It's also, we've said a lot of good stuff, but there's been lots of fights, there's been lots of stress, there's been trials and tribulations but it's never to a point where we're like, this is it. We're still together.” (24‐year‐old male survivor; in a relationship < 5 years)
Staying together [endorsed by couples in newer relationships or not married (*n* = 4 couples; 27%)]
“Obviously, I was devastated. [YA survivor] told me that she had stage four, so in my mind, I'm thinking, okay, my girlfriend, at the time, stage four, I'm thinking she's not gonna make it. I'm thinking she's gonna be a goner. So, I was like, all right, I'm gonna stick it out until she's gone. I thought that was the right thing to do, but she literally just went through 6 months of infusions, every 2 weeks for 6 months, and like she said, she's very lucky to be in remission now. But I thought she was gonna be a goner.” (34‐year‐old male partner; in a relationship < 5 years)

**Theme 3: Social support network challenges and opportunities**
Changing social networks (*n* = 8 couples; 53% of couples)
*Patient*: “Sometimes it's frustrating for us, I guess I can speak collectively because we share mutual ones [friends] too, but we're—you would also call us more mature. Maybe it’s expectations, but there are some people that just don't know what to say at all, and maybe they want to, or they don't know how to, and so they don't show up and that's a little disappointing, or their actions aren't—you're just—you're confused by it. So I mean, I would say sometimes you're surprised in the wrong way.” *Partner*: “And I think [patient] tends to get a little more frustrated by it, and I tend to be like, they're just immature, and we have to move on.” (24‐year‐old male survivor, 23‐year‐old female partner; in a relationship < 5 years)
Navigating family dynamics and support (*n* = 14 couples; 93% of couples)
“We've learned to navigate the differences in our families and how to have each other acclimate to that. Because my family is a very much a, tell us everything. That's what I'm expecting. So early on, it was kind of weird to be like, hey, so we just told you. We talked about it for a minute and a half, and now we're gonna keep eating dinner. That's kind of weird. And then to [partner], especially early on, it might've been overwhelming to share with my family. They're being supportive in their own way, but you have to stretch into that.” (36‐year‐old partner; married > 5 years)
Leaning on networks for support (*n* = 12 couples; 80% of couples)
*Partner*: “And then also, during all this time, we had a cruise planned for less than a week after he was diagnosed, so we were very uncertain if we were going to actually go on that cruise…But our kids were left with their grandparents, so it was—we knew that they were going to be taken care of.” *Survivor*: “It ended up, I think, being good, though, because we were able to talk openly.” (38‐year‐old male survivor, 37‐year‐old female partner; married > 5 years)
Types of helpful support
“Him [partner] and my dad were mainly the people that supported me all the time. His family too, they will support on the back with him helping with information, reading about—because I totally blocked my mind, try not read and not really go through like, people that had cancer and leukemia, those things. So I try really not to read anything about, so I can really dedicate, like being well mentally on the treatment. So that was like a big support for me as like, for my health and my point of view on that time.” (32‐year‐old female survivor; married < 5 years)

**Theme 4: Resource needs**
Tailored age‐appropriate resrouces and information (endorsed by all couples)
[*Talking about the packet of support information they received upon diagnosis]*. Survivor: “It's an 80 and a 90‐year‐old man sitting there having that talk about, well, if I'm not here anymore, you need to think about your will or your finances. And it is a different situation, obviously, if you're in your 30s going into cancer, versus in your 60s or 70s, and you've been married for 20 years, or you have adult children, and you think like, oh—it's a different priority in terms of what you're thinking of in your post‐cancer life. For us, it was okay, like we're going through all this, and now we have to start our lives—like, start our family. As opposed to, if you already have kids, or if you have other family issues, I guess. *Partner*: “It wasn't anything that would apply to him as a younger person, type deal.” (36‐year‐old male survivor; married > 5 years)
“Support groups, and not even just for the patient, but also for family members, the caregivers too, some people can really suffer. I think I was lucky because I had already experienced it with his mom, and then with his dad too, with his health issues. But it's a whole different thing when it's your husband going through it, or your boyfriend, or—at the time, fiancé … I know some of the local hospitals do support groups for the loved ones and caregivers. But it's the same thing, you're targeting an older popuation as opposed to young adults.” (37‐year‐old female partner; married > 5 years)

## 4 Dyadic Communication (Theme 1)

YA couples described a range of communication challenges and strengths (holding back, learning to talk about cancer, supporting one another) as important influences on their relationship and adjustment to cancer. Challenges were often exacerbated by concerns central to the “off‐time” nature of cancer within their relationship (e.g., fertility, loss of relationship goals, family responsibility, financial toxicity). Within this theme we identified three subthemes: positive communication, negative communication, and communication around difficult conversations.

### 4.1 Positive Communication

YA couples (*n* = 14 couples; 93%) described a shift in communication behaviors following the diagnosis, regardless of pre‐cancer communication styles. *“You're not beating around the bush anymore.”* Reported one YA partner, describing their communication as more *“blunt…direct.”* Specifically, cancer necessitated regular check‐ins between partners, discussing symptoms, treatment plans and appointments, and feelings and emotions. However, the nature of their newer, less established relationships presented a learning curve in how to best support one another, with couples mentioning that communication often got better over time and made their relationship stronger.

### 4.2 Negative Communication

YA couples (*n* = 10 couples; 67%) mentioned engaging in unsupportive communication behaviors, often induced by uncertainty, stress, and navigating a life‐threatening situation at an unforeseen time. Often YA couples realized that the negative communication strains on their relationships were a means of protecting one another. Feeling “not understood” by their partners was also commonplace. YA couples are most often jointly experiencing chronic illness for the very first time, navigating new physical limitations, and leaning into understanding the long‐term effects of cancer diagnosis and subsequent treatment.

### 4.3 Difficult Conversations

This subtheme surrounding difficult conversations was discussed by 60% of YA couples (*n* = 9 couples), with conversations mainly centered around fertility and mortality. Fertility was a primary concern for YA couples who were in the beginning stages of starting a family or thinking about growing their family (*n* = 6 couples; 40%), adding another medical hardship on top of dealing with cancer. Conversations around fertility focused on wishing they had more information in the beginning about the potential for infertility, feeling that this was glossed over in the treatment planning process. Conversations about mortality (*n* = 5 couples; 33%) sometimes resulted in avoidance of discussing the topic with one's partner and sadness about facing premature mortality.

## 5 Relationship Changes (Theme 2)

All YA couples discussed challenges and strengths arising from their cancer experiences. This theme resulted in three subthemes: shift in pre‐cancer responsibilities, strengthening of the relationship, and staying together.

### 5.1 Shift in Pre‐Cancer Responsibilities

Cancer evoked challenges for most YA couples (> half of all couples) that required a shift in their day‐to‐day routines. YA couples reflected on relationship changes rooted in adapting to the YA survivors' change in physical capabilities, medical obligations, and associated psychological concerns.

### 5.2 Strengthening of the Relationship

More than half of couples were resilient in coping with these cancer‐related challenges and even reported a growth in the foundation of their relationship as a result. Notwithstanding the challenges and stress, YA couples reported feeling a stronger connection to one another and talking more about everything. YA couples who were not currently married additionally described a stronger sense and trust and commitment within their newer relationships.

### 5.3 Staying Together

Fears of premature mortality and interruptions to a typical YA relationship experience (e.g. traveling, dating, working) incited conversations regarding staying together, primarily for couples in newer relationships or couples who were not married at the time (*n* = 4 couples; 27%). These conversations, often initiated by the YA survivor, focused on a discussion surrounding parting ways or staying together and figuring it out. In all instances couples decided to stay together with partners most often being the ones to contribute more greatly to that decision, stating that they are “not quitting” on the YA survivor, “that's not something I would ever do” and are “sticking around.”

## 6 Social Support Network Challenges and Opportunities (Theme 3)

We identified two subthemes for social network challenges: changing social networks and navigating family dynamics and support; and two subthemes for social network opportunities: leaning on networks for support and types of helpful support.

### 6.1 Changing Social Networks

Over the course of cancer diagnosis, treatment, and into survivorship, 53% of YA couples (*n* = 8 couples) experienced a change in their social networks (e.g., loss of friends or family members, less contact). A third of YA couples (*n* = 5 couples; 33%) who disclosed their cancer diagnosis in hopes of gaining support from certain friends or family were left feeling like the person they spoke with did not understand or care about them or their situation. On occasion, YA couples who had higher expectations of support provided by certain friends or family felt unsupported and wished that those members had provided more support by being both physically and emotionally available to them.

### 6.2 Navigating Family Dynamics and Support

Unique to the young adulthood time frame of cancer, differing familial support styles and perspectives emerged within interviews. Majority of YA couples (*n* = 14 couples; 93%) found it challenging to adapt to differing styles of familial support. Learning to adapt to differing styles of familial support was noted to be frustrating at times. However, YA couples described setting boundaries and adopting differing styles of communication when disclosing cancer to different family members. YA couples struggled to bring up cancer unsolicited with their social networks, many noting fears about worrying their friends and family. One couple described the interactions as *“burdening people with knowledge*”, another said that they *“didn't want to be the bearer of bad news*.” Others chose to avoid cancer as a topic to lessen the burden on their YA friends who were already busy with their lives, finding it challenging to ask for support and reported that they held back information. For those YA couples who engaged in cancer‐related conversations with their family members or friends they were either met with general support or mentioned conversations that felt socially constraining (e.g., lack of emotional disclosure). As a result, instead of keeping support networks updated on new cancer‐related details right away, YA couples often felt more comfortable if they had a plan for dealing with disclosing new information to their networks to lessen the intensity of information.

### 6.3 Social Network Opportunities

#### 6.3.1 Leaning on Networks for Support

Many couples (*n* = 12 couples; 80%) relied on support members, especially toward the early days of diagnosis, for emotional, informational, and tangible support. Having trustworthy individuals around taking care of children or to provide food provided more space for YA couples to find the time to talk with one another about cancer‐related topics, put plans in place, and be more emotionally available for each other.

### 6.4 Types of Helpful Support

Having a strong support network helped YA couples manage the emotional, physical, and logistical challenges of cancer. YA couples mentioned all types of support as being largely impactful. Receiving tangible support was most common among YA couples (*n* = 13 couples; 87%), followed by emotional support (*n* = 12 couples; 80% of couples), and informational support (*n* = 11 couples; 73% of couples). These supports gave couples a chance to focus on their mental and physical well‐being without worrying about cooking family meals, taking care of their children at that moment, or spending time researching everything about cancer alone.

## 7 Resource Needs (Theme 3)

Tailoring information and resources to fit the needs of YAs was particularly important to all YA couples. YA couples noted that most of the cancer‐related information they received was geared more toward older cancer patients with no guidance on how to find age‐appropriate support or information. When YA couples mentioned gaining support from YA‐focused resources, they generally had to search and advocate for them themselves.

YA couples were most interested in resources focused on navigating cancer as a young couple, financial toxicity, navigating insurance, sexual health concerns, emotional and physical challenges related to cancer, food insecurity, and cancer‐related decision‐making (e.g., fertility preservation). YA couples expressed interest in receiving resources in multiple ways, including through support groups, patient navigators, therapy, and/or information via QR codes.

## 8 Discussion

This present study explored communication and support‐focused experiences, needs, and concerns among YA couples. Through 15 semi‐structured qualitative interviews, we gathered four main themes of dyadic communication, dyadic relationship changes, support network challenges and opportunities, and resource needs of YA couples. Overall, our findings underscore the complexity of navigating a cancer diagnosis as a young adult couple and highlight the challenges and needs of this vulnerable population.

Our findings mirror dyadic relationship communication behaviors often seen in the adult couple literature (e.g., disclosure, holding back), but highlight notable differences in that communication challenges among YA couples were often exacerbated by concerns specific to the YA cancer population (e.g., fertility, premature mortality). YA couples noted needing time to figure out how best to communicate and support one another. This may be the first time in their young relationships jointly navigating a chronic life‐threatening illness which required new communication skills and knowledge to adapt to these changing relationship dynamics. The lack of communication, particularly around sensitive topics (e.g., mortality), often left YA couples to feel more burdened. However, when couples mentioned opening the lines of communication and sharing openly with one another, they felt closer and more connected to their partners and their relationships. This is critical as broader relationship models, such as the emergent distress model of marital development, suggests that an accumulation of negative communication behaviors (e.g., criticism, holding back) over time is responsible for relationship deterioration [37]. While young couples in newer relationships often exhibit low levels of negativity, the challenging cancer and post‐cancer landscape can alter pre‐cancer relationship dynamics.

Cancer among YA couples also resulted in relationship changes, with partners taking on increased responsibilities for household tasks and caregiving for the YA survivor. While most marriages are often resilient to these relationship changes, unique to YA couples are those in newer, non‐marital relationships, which necessitated conversations about staying together. While all non‐married couples were very committed to staying together, this also typically required delaying relationship goals and priorities (e.g., traveling, getting married). Despite these changes, YA couples described positive impacts, including a strengthened relationship, and for couples in newer relationships, a stronger sense of trust and security within their relationship.

Our findings also align with previous qualitative research among young adult couples coping with cancer focused on sexual health and family building [38, 39]. This small, yet impactful, body of work highlights the benefits of open communication, emotional and instrumental support, and working as a team as being critical to managing changes in young adult couples' relationships and sexual health. While there is no “one size fits all” solution, the overarching themes identified in our current work and previous young adult couple focused work may represent strategies that can be applied broadly to help young adult couples cope with and manage the challenges associated with cancer.

Navigating cancer as a young couple also presented unique challenges and opportunities centered on support from social networks, mainly family and friends. While YA couples implied a sense of gratefulness for the assistance of their families, often couples were learning how to get support from their families for the first time, including deciding what type of information they want to share, what type of support would be most helpful, and setting up boundaries surrounding family visits. Initiating conversations about cancer with support members was often difficult and felt out of the norm for YA couples. The uncertainty and fear of premature mortality made it challenging for young couples to bridge the topic of cancer with close friends and family, even when they needed and desired support.

Social support networks also served as a source of needed distraction from the day‐to‐day cancer experience. Consistent with stress‐buffering models of social support [22, 40], YA couples who reported being checked in on often by close friends and family mentioned being provided with a sense of comfort and normalcy in the wake of emotional distress. Support members also served to relieve some of the burden from YA couples through tangible support (e.g., cooking, cleaning, caring for children). This allowed YA couples critical time for open discussions about cancer‐related topics (e.g., planning, fears).

Also highlighted was the lack of resources specific to couples navigating cancer during young adulthood, which is consistent with prior young adult couple qualitative work [38]. When challenged with issues like sexual health and family planning, finances and insurance, lack of medical information, and navigating emotional and physical distress, YA couples mentioned needing to advocate for and seek out resources on their own. While couple‐identified resources (e.g., therapy, social support groups, social media) were overall beneficial for YA couples, this added role of advocacy added stress to their daily lives. Providing YA couples with trusted resources at the beginning of diagnosis and treatment may alleviate some of this advocacy role, and in turn reduce burden on the couple.

### 8.1 Implications

Positive dyadic and social network experiences strengthened relationships and seemed to promote more active engagement in necessary conversations. Through open styles of communication, both dyadically and externally with social network members, YAs were able to receive meaningful supports that reduced some of the stressors associated with cancer. Additionally, YA couples were proactive during their cancer journey, seeking out resources to aid their relationships, as well as addressing the emotional and physical distress of cancer. However, more work is needed to address the gaps in resources for navigating cancer as a young adult couple. Interventions and clinical support tailored to the unique experiences, needs, and concerns of YA couples have the potential to address these unmet needs and foster long‐term relationship satisfaction and well‐being among both members of the couple.

### 8.2 Limitations

This study is not without its limitations. Many of our participants expressed that they either previously or currently work in the medical field, allowing them access to certain medical knowledge that others may not have. Additionally, our sample population tends to be on the older side of the young adult range, therefore more research is needed to explore how younger adults and relationships communicate about cancer and gain social support. Given the qualitative nature of this study and the smaller sample size, we are limited in exploring potential differences based on demographic (e.g., gender), relationship [relationship type (e.g., same sex vs. different‐sex couples)], cancer‐related (cancer type, stage, treatment status) and/or contextual (e.g., level of support) factors. Future research exploring these subgroups may be one potential direction as these factors may influence communication patterns, relationship dynamics, and support needs among young adult couples coping with cancer. Lastly, our sample includes couples who stayed together during cancer diagnosis and subsequent treatment; thus, study findings might not be reflective of those couples who did not stay together after a cancer diagnosis.

## 9 Conclusions

This study is one of few that explore the in‐depth communication and social support‐focused needs and concerns surrounding YA couples, as well as gaps in resource needs. This work highlights YA couples needs that are central, and external, to the couple on communication and support in the face of a cancer diagnosis. Future research among YA couples should focus on developing evidence‐based interventions aimed at delivering meaningful information guide couple‐focused activities for navigating dyadic communication challenges and relationship changes, social support challenges and opportunities, and a focus toward sharing resources that are specific to young adult couples coping with cancer.

## Author Contributions

**Katie Darabos:** conceptualization, methodology, project administration, supervision, writing – original draft, formal analysis, visualization. **Sharon Manne, Katie Devine:** conceptualization, methodology, visualization, writing – review and editing. **Sean McHugh, Shannon Desbiens:** formal analysis, visualization, writing – original draft.

## Conflicts of Interest

The authors declare no conflicts of interest.
